# Re-evaluation of the definition of remission on the 17-item Hamilton Depression Rating Scale based on recovery in health-related quality of life in an observational post-marketing study

**DOI:** 10.1186/s12955-018-0838-6

**Published:** 2018-01-16

**Authors:** Jitsuki Sawamura, Jun Ishigooka, Katsuji Nishimura

**Affiliations:** 1 Department of Psychiatry, Tokyo Women’s Medical University, 8-1 Kawada-cho, Shinjuku-ku, Tokyo, 162-8666 Japan; 2 Ishigooka Hospital, 915 Hamano-cho, Chuo-ku, Chiba-shi, Chiba, Japan

**Keywords:** Depression, Remission, Health related quality of life, HAM-D17, SF-36, Cutoff value

## Abstract

**Background:**

Although a score of less than 7 for the 17-item Hamilton Depression Rating Scale (HAM-D17) has been widely adopted to define remission of depression, a full recovery from depression is closely related to the patient’s quality of life as well. Accordingly, we re-evaluated this definition of remission using HAM-D17 in comparison with the corresponding score for health-related quality of life (HRQOL) measured by the SF-36.

**Methods:**

Using the data for depressive patients reported by GlaxoSmithKline K.K. (Study No. BRL29060A/863) in a post-marketing observational study of paroxetine, with a sample size of *n* = 722, multivariate logistic regression was performed with the HAM-D17 score as a dependent variable and with each of the eight domain scores of HRQOL (from the SF-36) transformed into a binominal form according to the national standard value for Japan. Then, area under curve of receiver operating characteristic analyses were conducted. Based on the obtained results, a multivariate analysis was performed using the HAM-D17 score in a binomial form with HAM-D17 as a dependent variable and with each of the eight HRQOL domain scores (SF-36) as binominalized independent variables.

**Results:**

A cutoff value for the HAM-D17 score of 5 provided the maximum ROC-AUC at “0.864.” The significantly associated scores of the eight HRQOL domains (SF-36) were identified for the HAM-D17 cutoff values of ≥5 and ≤4. The scores for physical functioning (odds ratio, 0.473), bodily pain (0.557), vitality (0.379), social functioning (0.540), role-emotion (0.265), and mental health (0.467) had a significant negative association with the HAM-D17 score (*p* < 0.05), and HRQOL domain scores for HAM-D17 ≥ 5 were significantly lower compared with those for HAM-D17 ≤ 4.

**Conclusions:**

A cutoff value for HAM-D17 of less than or equal to 4 was the best candidate for indicating remission of depression when the recovery of HRQOL is considered. Restoration of social function and performance should be considered equally important in assessing the adequacy of treatment for patients with depression.

## Background

Guidelines for the treatment of major depressive disorders recommend that the primary therapeutic goal is to achieve remission in the acute phase of therapy [1, 2]. Traditionally, for the assessment of depression, the 17-item Hamilton Depression Rating Scale (HAM-D17) has been considered the gold standard [3, 4] with remission of depression indicated by a score of ≤7 [5–7]. However, depressive disorders also impair health-related quality of life (HRQOL) [8, 9]. Practically, it is widely recognized that both remission of depression symptoms and recovery of HRQOL are important in clinical treatment and research [10].

GlaxoSmithKline K.K. conducted a post-marketing clinical study to evaluate HRQOL using the Medical Outcome Study 36-Item Short-Form Health Survey scale (SF-36) in Japanese patients with depression treated with paroxetine. Overall, 12 weeks of treatment with paroxetine (20–40 mg/d) yield a significant improvement in both depression symptoms (HAM-D17) and HRQOL (SF-36). An investigation of the relationship between HAM-D17 scores and SF-36 scores was performed, and patients with higher degrees of improvement in depression symptoms tended to show higher degrees of improvement in HRQOL, as reported in an article related to the present study [11]. In the two SF-36 mental health dimensions of social functioning (SF) and role-emotional (RE), obtained scores were close to the level of the Japanese national norm when patients achieved remission of depression symptoms [12], which suggests a strong association between the remission of depression symptoms and recovery in HRQOL.

A HAM-D17 score of ≤7 was used to define remission of depression symptoms in the above post-marketing clinical study. While a HAM-D17 score of ≤7 has been widely adopted in clinical trials and research, little data support this threshold score from the perspective of HRQOL. Therefore, it is important to confirm the validity of this threshold score for remission of depression symptoms with respect to HRQOL for alignment with the primary therapeutic goal in the acute phase of therapy.

In this study, we investigated which symptom severity (HAM-D17) score was equivalent to recovery in HRQOL (SF-36 score) as indicators of remission. Through the analysis of raw data obtained from a post-marketing clinical study, we re-evaluated the definition of remission of depression to examine the validity and consistency of the existing definition (i.e., HAM-D17 score of ≤7) with respect to HRQOL.

Our specific objectives were as follows: (1) to investigate this definition of remission by comparison with the recovery in HRQOL for validity and consistency, (2) to examine the association of HRQOL domain scores (SF-36) with the severity of depression symptoms (HAM-D17 scores), and (3) to compare the depression symptoms (HAM-D 17 scores) of remitters (responders) and non-remitters (non-responders) using the recalculated cutoff score obtained in the present trial.

## Methods

### Data source and data management

#### Data sources for analysis

Raw data were obtained from a post-marketing clinical study of paroxetine in patients with depression or those experiencing depressive episodes, which included an evaluation of improvement by paroxetine of HRQOL (Study No. BRL29060A/863) [11, 13].

#### Data management

Raw data were obtained as anonymized patient data from GlaxoSmithKline K.K. Data were used and managed in compliance with a “Datasharing Agreement” with and authorized by GlaxoSmithKline K.K.

### Study design

This study is a reanalysis of the raw data from a post-marketing clinical study of paroxetine in patients with depression or those experiencing depressive episodes, which included an evaluation of improvement by paroxetine of HRQOL (Study No. BRL29060A/863).

### Demographic and clinical characteristics of patients

Height, weight, age, gender, onset of first depression or depressive episode, duration of the current episode, number of depressive episodes, depression diagnosis [14], past medical history, concurrent illness, treatment history, concurrent use of drugs, concurrent use of non-drug treatment (i.e., electroconvulsive therapy and cognitive behavioral therapy), comorbid anxiety disorders assessed by the Mini International Neuropsychiatric Interview (panic disorder, social anxiety disorder, obsessive-compulsive disorder, generalized anxiety disorder, posttraumatic stress disorder), dosing, and duration of paroxetine were evaluated.

### Efficacy

Total score on HAM-D17, score on each item of HAM-D17, state score and trait score on the State-Trait Anxiety Inventory (STAI) [15].

### Health outcome

The physical component summary (PCS) score on the SF-36 physical health dimension is composed of physical functioning (PF), role physical (RP), bodily pain (BP), and general health perceptions (GH).

The mental component summary (MCS) score on the SF-36 mental health dimension is composed of vitality (VT), SF, RE, and mental health (MH).

### Data analysis

#### Primary analysis

1) The scatter plot of each individual patient’s pairwise co-ordinates between HAM-D17 score and score on each HRQOL (SF-36) dimension was drawn. Then, the ability of HAM-D17 to identify patients who are in remission according to the recovery in HRQOL across the range of HAM-D17 cutoff scores by conducting receiver operating characteristic (ROC) analyses was examined. The HAM-D17 score showing the maximal level of agreement with the recovery in HRQOL was determined as the cutoff score of HAM-D17 for remission.

#### Secondary analysis

2) The cutoff value of HAM-D17 providing the maximal ROC-AUC was converted to a binominal form of “0 or 1.” A logistic multivariate analysis with HAM-D17 as the dependent variable and each of the eight HRQOL domains (SF-36) as binominalized independent variables was conducted to identify the relationship between them being associated with remitters (responders) or non-remitters (non-responders) defined using the HAM-D17 scores.

3) Mann-Whitney U tests were conducted to compare the domain subscores of HRQOL (SF-36) (PF, RP, BP, GH, VT, SF, RE, MH, PCS, and MCS) and STAI (the State Anxiety Scale and the Trait Anxiety Scale) between remitters (responders) or non-remitters (non-responders) using the recalculated cutoff score obtained in the present trial.

We used SPSS for Windows Version 20 [16] and Stata Release 13.0 [17] for statistical analysis and Microsoft Excel 2003 [18] for plotting the graph.

## Results

**Fig. 1:**
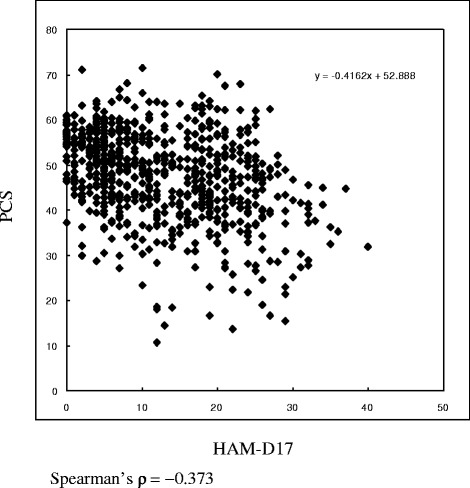
Scatter plot between HAM-D17 and PCS scores

**Fig. 2:**
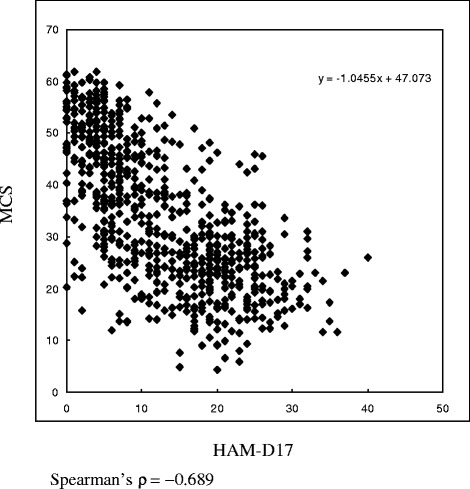
Scatter plot between HAM-D17 and MCS scores

Patients treated with paroxetine (maximum dose of 20–40 mg/d) were assessed over 12 weeks (0, 4, 8, and 12 weeks, where “0 week” means the subject dropped from the study before week 4 for any reason). The sample number (*n* = 722) for analysis included assessments at all time points for participating patients (*n* = 217). The scatter plots between HAM-D17 and PCS and MCS are shown in Figs. 1 and 2. For the sample of n = 722, a multivariate logistic regression was performed with the score of HAM-D17 as a dependent variable and each of the eight domain scores of HRQOL (SF-36) as independent variables (converted into binominal form as “0 or 1” according to the national standard value for Japan). By assigning the different integers of the HAM-D17 scores as cutoff values, ROC analyses were conducted and area under curve of ROC (ROC-AUC) was calculated (Table 1). A cutoff value of 5 for HAM-D17 provided the maximum ROC-AUC as “0.864.” After converting this cutoff score of HAM-D17 into the binominal form of “0 or 1” for HAM-D17 of ≥5 and ≤4, a multivariate logistic regression analysis with HAM-D17 as the dependent variable and each of the eight HRQOL domain scores (SF-36) as binominalized independent variables was performed. Then, the significantly associated scores of the eight HRQOL domains (SF-36) were identified (Table 1). Specifically, the six subscores of PF (odds ratio, 0.473), BP (0.557), VT (0.379), SF (0.540), RE (0.265), and MH (0.467) were negatively associated with HAM-D17. The Spearman’s ρ of the HRQOL subscores with HAM-D17 are presented in Table 2. All HRQOL subscores were negatively correlated with the HAM-D17 scores. On performing Mann-Whitney U tests, it was observed that all respective subscores of HRQOL at HAM-D17 ≤ 4 are significantly higher than those at HAM-D17 ≥ 5, and State/Trait-Anxiety of STAI for HAM-D17 ≤ 4 were significantly lower than those for HAM-D17 ≥ 5 (Tables 3 and 4).

**Table 1:** Results of ROC-AUC obtained by multivariate logistic regression

HAM-D17 Cut-off value	ROC-AUC	95% Confidence Interval	Selected Scores of HRQOL
1	0.868	0.813–0.942	BP, VT
2	0.854	0.799–0.909	BP,VT,SF,RE
3	0.828	0.779–0.877	VT,SF,RE
4	0.848	0.808–0.887	BP,SF,RE,MH
5	0.864	0.831–0.898	PF,BP,VT,SF,RE,MH
6	0.852	0.819–0.885	PF,VT,SF,RE,MH
7	0.837	0.803–0.870	PF,VT,SF,RE,MH
8	0.844	0.813–0.875	PF,BP,VT,SF,RE,MH
9	0.837	0.806–0.868	PF,BP,VT,SF, RE,MH
10	0.834	0.803–0.864	PF,BP,SF,RE,MH
11	0.836	0.806–0.866	PF,BP,VT,SF,RE,MH
12	0.830	0.800–0.860	PF,BP,SF,RE,MH
13	0.820	0.789–0.850	PF,BP,SF,RE,MH
14	0.825	0.795–0.855	PF,BP,SF,RE,MH
15	0.820	0.790–0.850	PF,BP,RE,MH
16	0.813	0.782–0.843	PF,BP,SF,RE,MH
17	0.794	0.762–0.826	PF,BP,SF,RE,MH
18	0.790	0.758–0.822	PF,BP,RE
19	0.793	0.761–0.825	PF,BP,SF,RE
20	0.789	0.756–0.823	PF,SF,RE
21	0.792	0.758–0.826	PF,SF,RE
22	0.779	0.732–0.817	PF,RE
23	0.773	0.733–0.813	

Abbreviation: *HRQOL* health-related quality of life, *PF* physical functioning, *RP* role physical, *BP* bodily pain, *GH* general health perceptions, *VT* vitality, *SF* social functioning, *RE* role-emotional, *MH* mental health

The multivariate logistic regression with the HAM-D17 score as a dependent variable and with each of the eight domain scores of HRQOL (SF-36) as independent variables was conducted, and the maximal ROC-AUC of 0.864 was obtained. In addition, based on the cutoff value, by converting the HAM-D17 score into the binominal form of “0 or 1,” a multivariate analysis with HAM-D17 as a dependent variable and with each of the eight HRQOL domain scores (SF-36) as binominalized independent variables was performed. Then, the significantly (*p* < 0.05) associated scores of the eight HRQOL domains (SF-36) were identified. Specifically, after converting this cutoff score of HAM-D17 into the binominal form of “0 or 1” for HAM-D17 scores of ≥5 and ≤4, the domain scores of PF, BP, VT, SF, RE, and MH are significantly negatively correlated with HAM-D17

**Table 2:** Spearman’s ρ among HAM-D17 and subscores of HRQOL

	PCS	MCS	PF	RP	BP	GH	VT	SF	RE	MH
HAM-D17	−0.3732	−0.6890	−0.4656	−0.4542	−0.3700	−0.5325	−0.6891	−0.5488	−0.5813	−0.7049

(*p* < 0.001)

Abbreviations: *PCS* Physical component summary, *MCS* Mental component summary, *PF* physical functioning, *RP* role physical, *BP* bodily pain, *GH* general health perceptions, *VT* vitality, *SF* social functioning, *RE* role emotional, *MH* mental health

The Spearman’s ρ of the HRQOL subscores with HAM-D17 are presented. All HRQOL subscores (SF-36) have a significantly negative correlation with HAM-D17

**Table 3:** Mann–Whitney U tests in subscores of HRQOL

	PCS	MCS	PF	RP	BP	GH	VT	SF	RE	MH
Total	47.57 ± 9.78	33.72 ± 13.45	86.28 ± 15.95	51.87 ± 42.24	67.98 ± 25.64	50.29 ± 20.49	40.13 ± 23.74	60.86 ± 26.76	39.52 ± 42.23	49.63 ± 23.83
HAM-D17 ≥ 5	46.26 ± 10.00	30.16 ± 11.85	84.23 ± 16.53	44.02 ± 41.42	64.38 ± 25.58	46.14 ± 19.19	33.97 ± 21.08	55.16 ± 25.47	29.46 ± 37.76	43.70 ± 21.72
HAM-D17 ≤ 4	52.79 ± 6.69	47.90 ± 9.53	94.41 ± 9.87	81.10 ± 29.00	82.31 ± 20.37	66.79 ± 16.94	64.62 ± 16.96	83.53 ± 18.50	79.54 ± 34.74	73.26 ± 15.90

*p* < 0.001

Abbreviations: *PCS* Physical component summary, *MCS* Mental component summary, *PF* physical functioning, *RP* role physical, *BP* bodily pain, *GH* general health perceptions, *VT* vitality, *SF* social functioning, *RE* role emotional, *MH* mental health

In Mann-Whitney U tests, all respective scores of HRQOL for HAM-D17 ≤ 4 are significantly higher than those of HAM-D17 ≥ 5

**Table 4:** Mann–Whitney U tests in subscores of STAI

	S-Anxiety	T-Anxiety
Total	49.03 ± 12.04	52.62 ± 13.13
HAM-D17 ≥ 5	51.93 ± 10.92	55.91 ± 11.45
HAM-D17 ≤ 4	37.50 ± 9.01	39.51 ± 11.05

*p* < 0.001

Abbreviations: *S-Anxiety* the State Anxiety Scale, *T-Anxiety* the Trait Anxiety Scale

In Mann-Whitney U tests, the two scores of STAI for HAM-D17 ≤ 4 are significantly lower than those of HAM-D17 ≥ 5

## Discussion

As is well known, severe depression often causes severe impairment of quality of life in patients with depression. In the present study, a cutoff value of 4 or lower (≤4) on HAM-D17 provided the maximal ROC-AUC or maximal level of agreement with HRQOL recovery. At this cutoff value, six subscores of HRQOL (SF-36), namely PF and BP (in the physical health dimension) and VT, SF, RE, and MH (in the mental health dimension), were identified with significantly negative association with the HAM-D17 scores around 4–5 points. This result was consistent with an existing report, which states that SF and RE scores return to close to the level of the Japanese national norm in Japanese patients with depression who are remitters [12]. The significant relation of BP with HAM-D17 was understandable because depression with pain often harms a patient’s QOL, and alleviation of pain is one of primary symptoms to be ameliorated in patients with depression.

Since 1991, a score of ≤7 has been a consensus in previous reports on the HAM-D17 cutoffs for remission [19]. The American College of Neuropsychopharmacology (ACNP) recommended a score of ≤7 or ≤5 [7, 20]. On the contrary, Zimmerman et al. reported that the cutoff score of 7 on HAM-D17 could be high [21–23], and others supported similar results [24, 25]. Zimmerman also reported that remitters (HAM-D17 ≤ 7) had heterogeneity in their symptoms and that the lower cutoff could be more suitable for remission if patients’ social functionality was considered [21–23]. Similar results were observed in the Montgomery Åsberg Depression Rating Scale (MADRS) [26], where patients with depression having lower scores (≤4 vs 5–9) appeared to have better global functioning [27]. Romera et al. proposed a similar suggestion based on the Social and Occupational Functioning Assessment Scale [28, 29]. A similar result was reported for functionality [30]. Furukawa et al. reported that this gap was due to the fact that functional recovery occurs after clinical recovery [31]. In addition, for the two scores of STAI, the differences based on the cutoff scores of HAM-D17 (≥5 and ≤4) indicated recovery from anxiety at this level as well. The present study investigated the relationship between depression and HRQOL which may have contributed as a reference for psychotherapeutic interventions. However, it revealed the need of further studies in this field.

Given this literature, the identification of a cutoff score of ≤4 in our study is consistent. A 3-point gap between the re-estimated cutoff score of ≤4 and the traditional cutoff score of ≤7 was observed. This might imply that the recovery of HRQOL lags behind that of depressive symptoms (HAM-D17), and that additional time is required for patients’ SF and QOL to reflect their HAM-D17 score. Moreover, the assessment of patients’ functional status is vital for precise estimation of QOL. In this regard, a “cutoff value of ≤4 for HAM-D17” is not inconsistent with this suggestion and also supports the propositions of Zimmerman and others.

Limitations in our approach should be noted. First, the analysis of the present study was performed retrospectively based on a specific population with clear limitations; however, the intent-to-treat method was used. Data might be biased because of our post hoc approach. In addition, the interpretation of the results of this study could not be applied to other conditions, countries, species, prescription, dietary, climate, or culture either simply or directly. These factors should be considered in the context of the respective treatment. We expect that these limitations will be remedied in a future study.

Second, the clinical course of patients with depression could have been influenced by the prescription of paroxetine. The data for the clinical course for assessment were not obtained in a series of patients undergoing a natural course of depression without drug (drug-naïve patients with depression). At the very least, a prospective observational study under prescription is needed.

Third, the sample (*n* = 722) in this study did not comprise completely independent data, because there are several observations for most patients (*n* = 217). The lack of independence of the sample data set might affect the value of the cutoff score for HAM-D17. However, randomly obtained data are considered less meaningful in determining the turning point in the clinical course of depression. Thus, this trade-off might be necessary.

Fourth, for the results of the logistic regression of HRQOL, the level of reproducibility for the selection of the six subscores of PF, BP, VT, SF, RE, and MH that were negatively correlated with HAM-D17 is unclear. In addition, to identify specifically associated symptoms with the HAM-D17 scores around 4–5 points (HAM-D17 cutoff values of ≥5 or ≤4), six symptoms cover almost the entire score of the HRQOL. Focusing on specific domain scores of QOL might not always provide consistent information.

Fifth, the entire data set is dependent on Japanese characteristics, which could be heterogeneous and could also introduce unexpected biases or tendencies in the results. Specific geographic locality and population are necessary tradeoffs in research. Therefore, any generalization from these results should be used with caution.

Additional investigations using more rigorous methodology in future studies to confirm these findings will be necessary.

## Conclusions

The cutoff value for HAM-D17 score of less than or equal to 4 (≤4) was one of the candidates for the remission of depression if HRQOL recovery is considered. The recovery of social function and performance are equally important for patients with depression for the remission of depressive symptoms.

## Abbreviations

ACNP: American College of Neuropsychopharmacology
BP: Bodily pain
GH: General health perceptions
GSK: GlaxoSmithKline K.K.
HAM-D: Hamilton Depression Rating Scale
HAM-D17: 17-item Hamilton Depression Rating Scale
HRQOL: Health-related quality of life
MADRS: Montgomery Åsberg Depression Rating Scale
MCS: Mental component summary
MH: Mental health
PCS: Physical component summary
PF: Physical functioning
RE: Role-emotional
ROC: Receiver operating characteristic
ROC-AUC: Area under curve of receiver operating characteristic
RP: Role-physical
SF: Social functioning
SF-36: 36-Item Short-Form Health Survey scale
SOFAS: the Social and Occupational Functioning Assessment Scale
S-Anxiety: the State Anxiety Scale
STAI: the State-Trait Anxiety Inventory
T-Anxiety: the Trait Anxiety Scale
VT: Vitality
